# A cross-database bibliometric analysis of rapid eye movement sleep behavior disorder in Parkinson’s disease research

**DOI:** 10.3389/fnagi.2025.1744493

**Published:** 2026-01-15

**Authors:** Shengjie Du, Xijiang Tian, Rui Ren, Luya Shi

**Affiliations:** 1 Department of Nursing Administration, Municipal Hospital Affiliated to Taizhou University, Taizhou, Zhejiang, China; 2 Postgraduate School of Nursing, Sehan University, Yeonggam, Republic of Korea; 3 Office of the University, Weifang University of Science and Technology, Weifang, Shandong, China

**Keywords:** bibliometric, CiteSpace, nonmotor symptoms, Parkinson’s disease, REM Sleep Behavior Disorder, VOSviewer

## Abstract

**Background:**

Rapid eye movement sleep behavior disorder (RBD) is increasingly recognized as both a prodromal marker and a significant predictor of Parkinson’s disease (PD) progression. Despite a surge in related research, a comprehensive bibliometric evaluation summarizing the field’s development, key contributors, and thematic evolution remains lacking. This study aimed to uncover the knowledge structure and emerging frontiers in RBD-related PD research through bibliometric analysis.

**Methods:**

On September 1, 2,025, an extensive literature search was conducted in the Web of Science Core Collection and Scopus databases using standardized RBD-related PD keywords. Bibliometric analysis and knowledge mapping were performed with CiteSpace, VOSviewer, and R software.

**Results:**

A total of 2,887 publications were identified, research output has increased steadily since 2013. Keyword co-occurrence and clustering analyses revealed three primary research directions: (1) longitudinal studies of RBD as a prodromal manifestation of synucleinopathies, (2) biomarker discovery for early diagnosis and disease monitoring, and (3) clinical interventions targeting sleep disturbances and neuroprotection. Notably, recent research trends emphasize non-motor symptoms of PD, overlapping mechanisms with Lewy body disease, and the application of advanced neuroimaging and digital sleep-monitoring technologies. Additionally, emerging keywords highlight biomarkers, gender differences, melatonin as focal points.

**Conclusion:**

This bibliometric analysis provides a systematic overview of the RBD-related PD research landscape. It underscores the field’s shift from clinical observation to mechanistic exploration and translational application. These findings may guide future studies aimed at improving early detection and developing individualized therapeutic strategies for patients with RBD and PD.

## 1 Introduction

Rapid eye movement sleep behavior disorder (RBD) is a prevalent non-motor symptom of Parkinson’s disease (PD), serving as both a prodromal manifestation and an early predictor of PD progression (Schutz et al., 2022; Byeon, 2020; Benbir-Senel et al., 2024; Berg et al., 2021). Approximately 50% of patients with PD exhibit RBD, compared to a prevalence of 0.38 to 1.15% among individuals over 60 without neurological conditions (Benbir-Senel et al., 2024). Furthermore, around 6% of idiopathic RBD (iRBD) cases progress to neurodegenerative diseases each year, particularly PD and dementia with Lewy bodies (DLB), with a conversion rate exceeding 73.5% over 12 years (Benbir-Senel et al., 2024; Borghammer and Van Den Berge, 2019; Postuma et al., 2019; Galbiati et al., 2019; Baumann-Vogel et al., 2020; Xie et al., 2021). RBD is characterized by vivid, often violent dreams during REM sleep, accompanied by abnormal motor behaviors, including vocalizations, limb flailing, punching, kicking, and even self-injury or injury to bed partners (Schutz et al., 2022; Di Folco et al., 2024). These behaviors severely disrupt sleep quality and negatively impact the quality of life for both patients and caregivers (Schutz et al., 2022; Di Folco et al., 2024). PD individuals with RBD are typically characterized by a more aggressive disease progression and are more frequently associated with an akinetic-rigid motor phenotype. In addition, these patients exhibit a higher burden of non-motor symptoms, including constipation, hallucinations, depression, and cognitive impairment, which collectively contribute to increased disease burden and higher mortality risk (Berg et al., 2021; Xie et al., 2021; Di Folco et al., 2024; Marafioti et al., 2023; Assogna et al., 2021). The global aging population has brought greater focus to the early diagnosis and management of RBD as a key component of PD prevention and personalized intervention strategies.

Research indicates that RBD is closely linked to the abnormal accumulation of alpha-synuclein in the brainstem, which disrupts the inhibition of motor activity during REM sleep, leading to dream enactment (Di Folco et al., 2024). Structural and functional alterations in various brain regions, including the brainstem nuclei, basal ganglia, frontal lobe, and occipital lobe, have been observed in patients with RBD, with these changes lying intermediate between those seen in healthy controls and patients with PD (Valli et al., 2021; Yang et al., 2020; Matzaras et al., 2022). Predictors for the conversion of iRBD to PD include impaired motor function, constipation, orthostatic hypotension, hyposmia, mild cognitive impairment, and color vision abnormalities (Benbir-Senel et al., 2024; Xie et al., 2021; Di Folco et al., 2024; Marafioti et al., 2023; Assogna et al., 2021; Wang et al., 2022; Solla et al., 2023). Polysomnography (PSG) remains the gold standard for diagnosing RBD, while neuroimaging techniques such as positron emission tomography (PET), single-photon emission computed tomography (SPECT), magnetic resonance imaging (MRI), and biomarker analysis are pivotal for early detection of neurodegenerative changes in RBD (Valli et al., 2021; Valli et al., 2022; Cesari et al., 2022). Current treatment approaches include pharmacological options, such as high-dose melatonin and low-dose clonazepam, and non-pharmacological measures like creating a safe sleep environment and utilizing bed alarm systems, which have shown efficacy in reducing the frequency and severity of harmful behaviors and suppressing unpleasant dreams (Gilat et al., 2022; Yan et al., 2021; Taximaimaiti et al., 2021). However, no effective interventions currently exist to delay or prevent the progression of iRBD to PD or other neurodegenerative disorders. Consequently, research into early diagnostic methods and interventions aimed at delaying or preventing the conversion of RBD to PD or other neurodegenerative conditions has gained substantial momentum, with a marked increase in the volume of related literature. Nevertheless, a systematic evaluation of the development trends, research hotspots, and the evolution of this field remains absent.

Bibliometrics, as a quantitative approach for analyzing scientific literature, provides a macro-level perspective that uncovers the knowledge structure, research hotspots, and developmental trends within a field, thereby enhancing researchers’ understanding of the domain (Donthu et al., 2021; Zupic and Čater, 2015; Ellegaard and Wallin, 2015). Knowledge mapping and hotspot visualization analysis, essential tools in bibliometric research, facilitate the intuitive representation of knowledge networks and hotspot distributions in a given area (Moral-Muñoz et al., 2020; Linnenluecke et al., 2020). In recent years, bibliometric techniques have been extensively applied in medical research, yielding valuable insights into topics such as accidental falls, speech disorders, swallowing difficulties, biomarkers, and acupuncture treatments in patients with PD (Shi and Yih, 2024; Zhao et al., 2024; Sun et al., 2024; Pan et al., 2024; Cinpolat and Akar, 2023). Despite its utility, bibliometric analysis has not yet been applied to RBD-related PD research. Thus, this study aims to systematically examine the knowledge map and hotspot visualization of RBD within the context of PD using bibliometric methods. The objectives are to identify the core themes and research directions, trace the evolution of research focus, and highlight emerging frontiers in the field. The findings will establish a comprehensive knowledge framework for researchers and provide theoretical support for future investigations into RBD-related PD.

## 2 Materials and methods

### 2.1 Data collection

**Figure 1:**
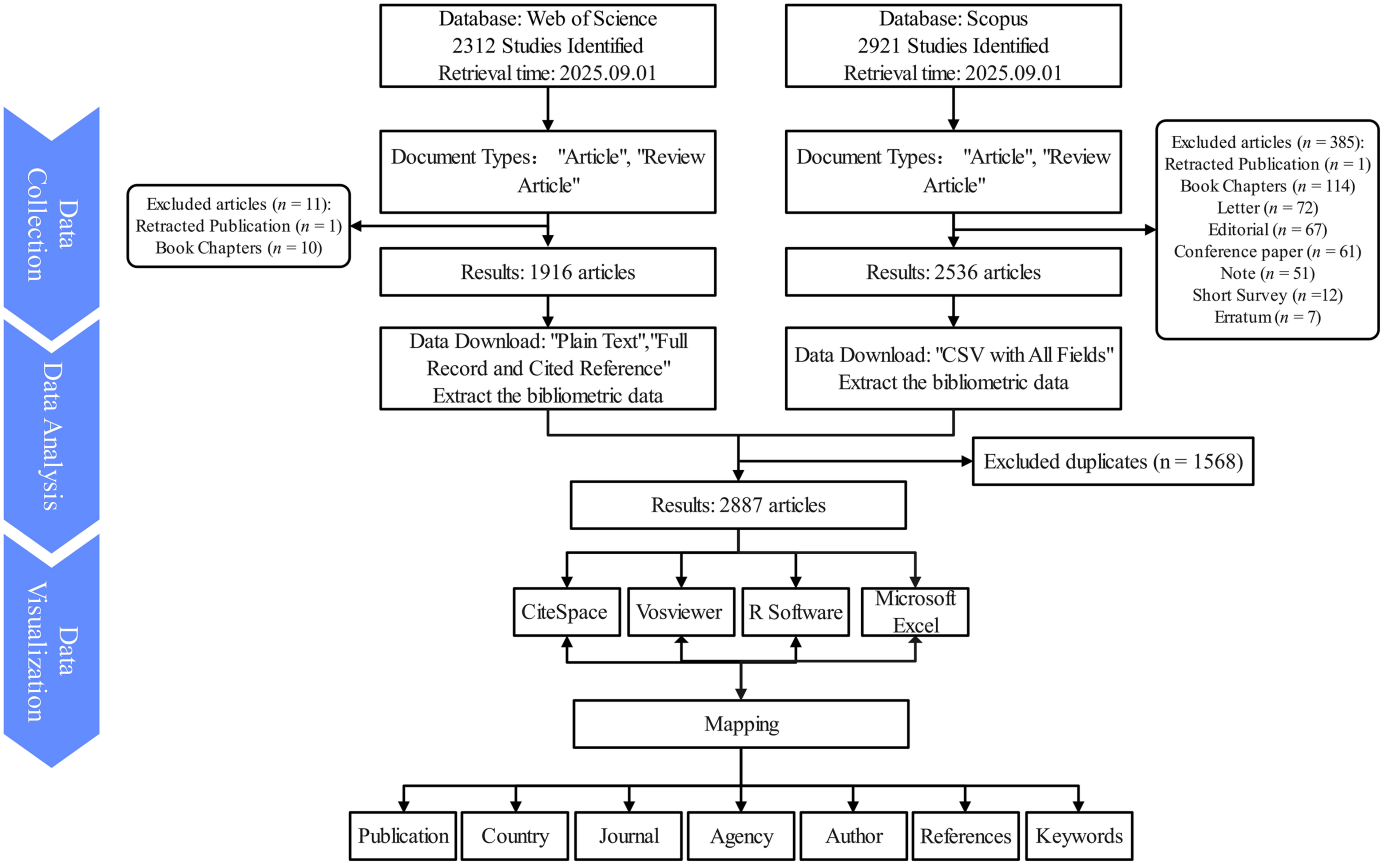
Inclusion and exclusion processes of research on REM Sleep Behavior Disorder in Parkinson’s disease.

A bibliometric analysis of RBD-related PD publications was performed using the Web of Science Core Collection (WoSCC) and Scopus databases, both recognized for their authoritative and comprehensive coverage of globally influential academic literature. To maximize search accuracy and completeness, keywords were initially compiled from a preliminary literature review and subsequently refined using PubMed MeSH terms. The search was conducted on September 1, 2025, encompassing all records from the inception of each database up to the specified date. The final search query in the WoSCC was formulated as: TS = (“REM Sleep Behavior Disorder*” OR “REM Behavior Disorder*” OR “Rapid Eye Movement Sleep Behavior Disorder*”) AND TS = (parkinson*). In WoSCC, 2,312 publications were initially identified, with subsequent filtering to include only “Article” and “Review Article” document types while excluding retracted publications, resulting in a final dataset of 1,916 articles (Figure 1). In Scopus, the search was performed using “Article title, Abstract, Keywords,” the search query was formulated as: TITLE-ABS-KEY (“REM Sleep Behavior Disorder*” OR “REM Behavior Disorder*” OR “Rapid Eye Movement Sleep Behavior Disorder*”) AND TITLE-ABS-KEY (parkinson*). In Scopus, 2,921 records were initially identified, and after restricting to “Article” and “Review” types 2,536 articles were retained. The WoSCC search results were exported in Plain Text File format, including Full Records and Cited References, while the Scopus records were exported in “CSV with All Fields.” Records retrieved from the WoSCC and Scopus were combined and managed using EndNote 2025. Duplicate records were removed through a two-step procedure. First, EndNote’s automatic duplicate detection function was applied to identify records with identical Digital Object Identifiers (DOIs). Second, a manual screening was performed to identify residual duplicates based on article titles, author(s), and publication years. When duplicate records were identified across databases, the WoSCC version was retained, as its metadata are generally more standardized and more compatible with bibliometric analysis software. This approach ensured the accuracy and consistency of the final dataset, resulting in 2,887 unique publications for subsequent analysis.

To ensure the reliability of the data, two researchers independently conducted the literature search, document download, and bibliometric data extraction on the same day. Any discrepancies were resolved through consensus discussions with a third researcher.

### 2.2 Data analysis

CiteSpace was employed to consolidate keyword synonyms, and remove irrelevant terms, ensuring clarity and consistency in subsequent keyword analyses. Following this, bibliometric and knowledge mapping analyses were conducted using VOSviewer 1.6.20, Bibliometrix, CiteSpace 6.3. R1, R software, and Microsoft Excel 2019.

#### 2.2.1 VOSviewer

VOSviewer, a prominent software in bibliometric analysis, was utilized to construct networks of author collaborations and keyword co-occurrences (van Eck and Waltman, 2010). In these visualizations, nodes and connecting lines of varying sizes and colors represent the frequency of occurrence and the strength of relationships between authors and keywords, respectively. The minimum number of occurrences was set to 5 for keywords and 10 for authors. These thresholds were selected to balance network interpretability and information retention and were applied consistently across analyses. Network normalization was performed using the association strength method, which is recommended for co-occurrence analyses in VOSviewer. Items not meeting the predefined thresholds were excluded automatically by the software.

#### 2.2.2 CiteSpace

CiteSpace, developed by Professor Chaomei Chen, is a Java-based bibliometric and visualization tool used to analyze structural and temporal dimensions of countries, institutions, authors, journals, keywords, and co-cited literature (Chen et al., 2010; Chen and Song, 2019; Chen, 2006). The visualizations depict relationship strength through the thickness of connecting lines, while node size indicates the frequency of occurrence. The analysis parameters were configured with the Time Slice set to 1 year per slice and the Selection Criteria set to g-index: *k* = 25. To reduce network complexity and highlight the most significant connections, the Pathfinder pruning algorithm was applied, together with pruning of sliced networks where appropriate. Keyword and reference clustering was conducted using the log-likelihood ratio (LLR) algorithm, and cluster labels were generated automatically by CiteSpace.

#### 2.2.3 Bibliometrix

Bibliometrix (R package, Biblioshiny 5.0) was used primarily for descriptive analyses and trend visualization. Default parameter settings were applied unless otherwise specified, including standard preprocessing of metadata and frequency-based inclusion thresholds.

## 3 Results

### 3.1 Annual publications and trend

**Figure 2:**
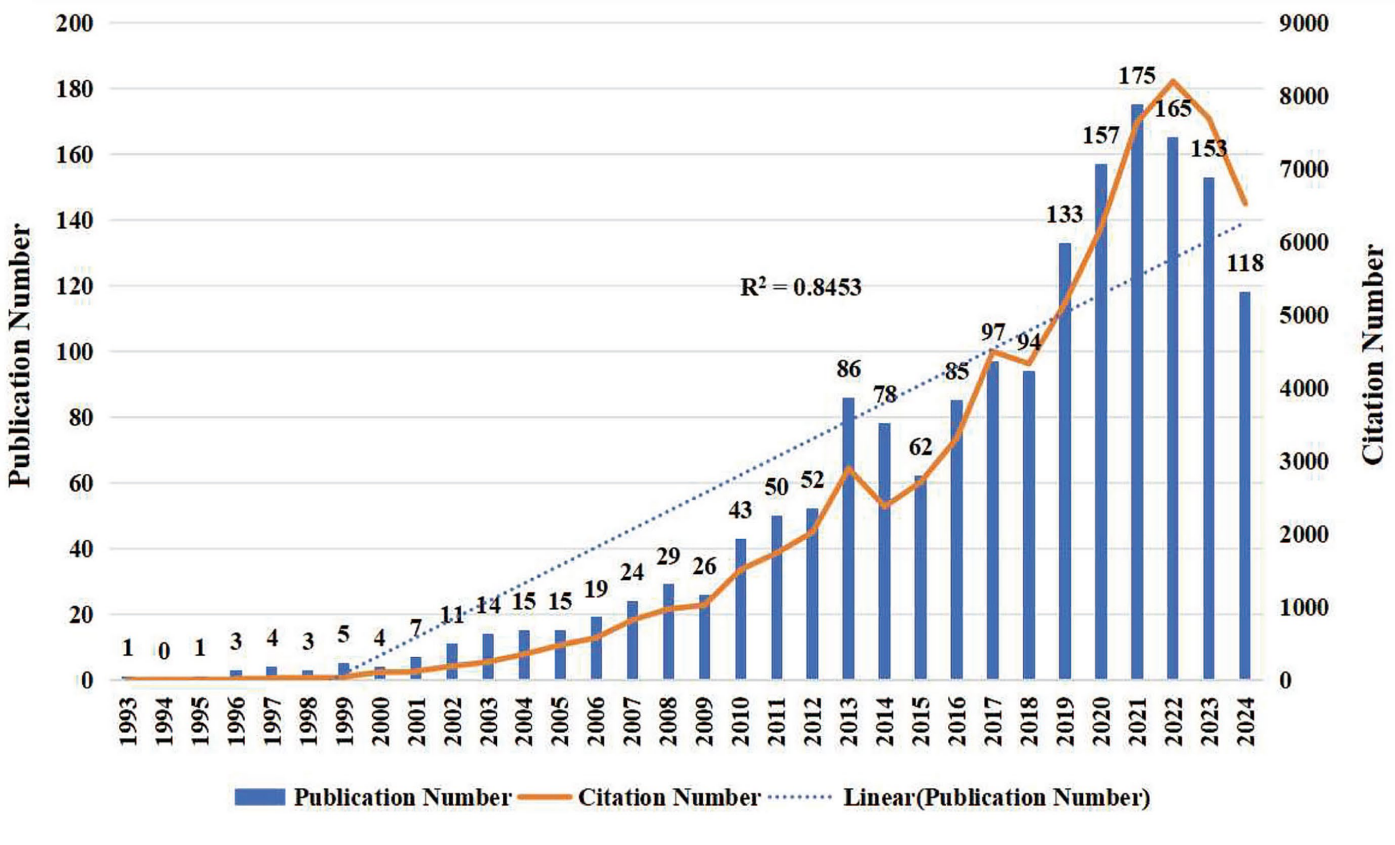
Annual publication and citation growth trend of research on REM Sleep Behavior Disorder in Parkinson’s disease. Temporal trends in annual publications and citations related to REM Sleep Behavior Disorder in Parkinson’s disease from 1993 to 2025. The bar chart represents the annual number of publications, while the solid line represents the yearly citation number.

Analyzing the annual publication volume and trends in a research field provides insights into its academic impact and global research dynamics (Chen and Song, 2019). As of September 1, 2025, the WoSCC and Scopus databases indexed 2,887 publications on RBD in PD, with a total of 76,730 citations, yielding an average of 41.49 citations per article and an H-index of 117. The earliest publication appeared in 1993, with the highest publication volume recorded in 2024, totaling 301 articles. The annual publication growth rate has averaged 17.17%, with a correlation coefficient (R^2^) of 0.87, indicating a strong upward trend. Notably, the growth rate has accelerated significantly since 2010 (Figure 2), with three major peaks in publication volume observed in 2013, 2021, and 2024. Citation activity began to rise more sharply from 2018 onward, following an almost exponential trajectory, with citation peaks occurring in 2013, 2017, and 2022.

### 3.2 Analysis of countries

**Figure 3:**
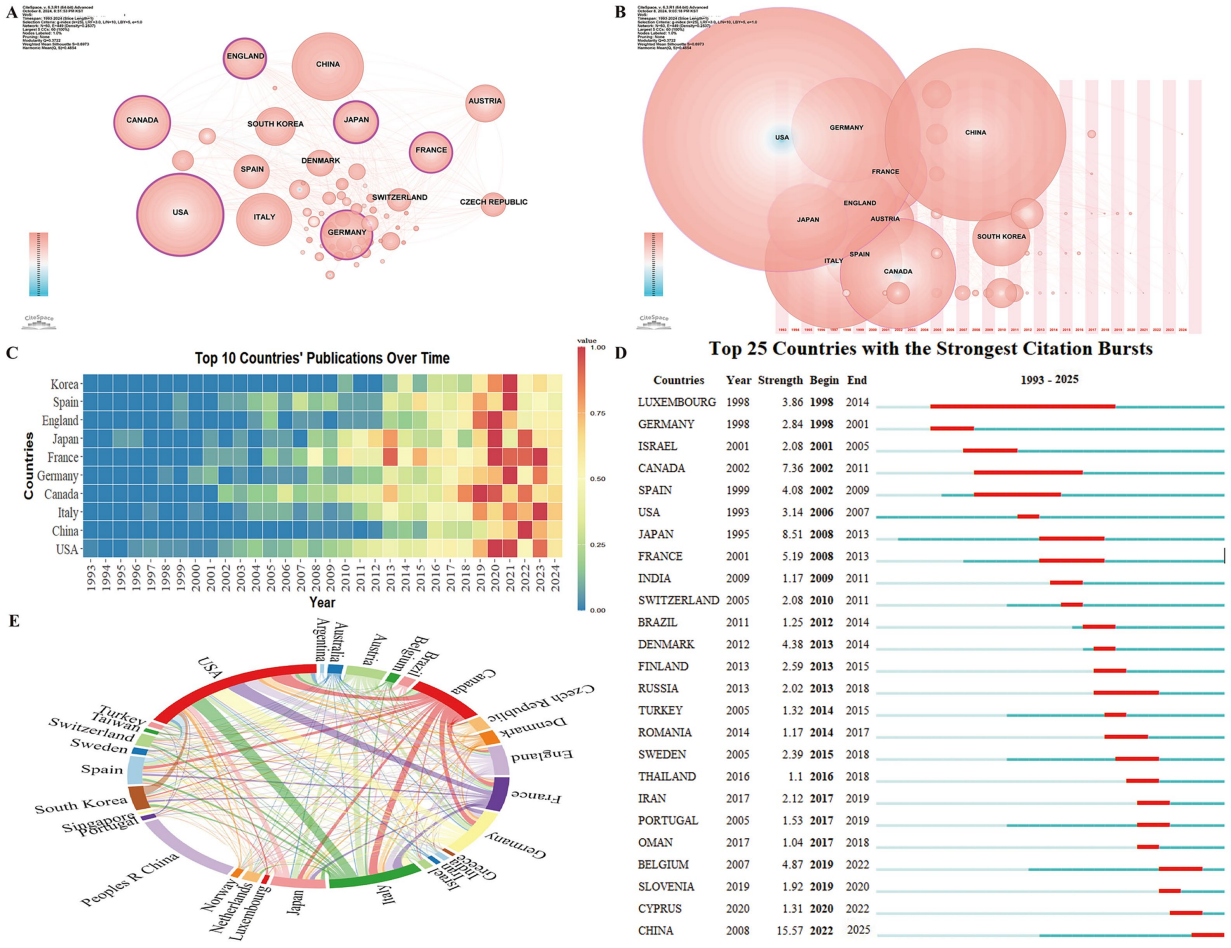
Geospatial analysis of research output on REM Sleep Behavior Disorder in Parkinson’s disease. **(A)** Co-occurrence network of contributing countries. Node size corresponds to the frequency of co-occurrence, while nodes encircled in purple indicate high centrality (≥ 0.1), signifying influential bridging roles. **(B)** Co-occurrence network showing the timezone of contributing countries. The horizontal axis represents the year of the country’s first published article, with node size indicating the number of articles published. Blue nodes signify earlier publications, while pink nodes indicate more recent ones. Overlapping colors reflect multiple publications within a given year, with a higher count forming rings, symbolizing continuous and extensive publication activity over time. **(C)** Temporal trends in annual publication output from the top 10 most prolific countries. **(D)** Top 25 countries with strongest citation bursts. **(E)** Collaboration network among countries, where link thickness indicates the strength of collaboration.

Country analysis highlights the leading contributors in RBD-related PD research and evaluates patterns of international collaboration. Research output spans 61 countries, with a country centrality analysis conducted using CiteSpace (Figure 3A). Centrality values indicate a country’s pivotal role within the field, with six nations exhibiting high centrality: the USA (0.35), Japan (0.27), England (0.25), Germany (0.19), Canada (0.16), and France (0.14), signifying their leadership roles in advancing RBD-related PD research.

The country-specific publication time-zone chart illustrates the initiation year and annual publication trends for each country (Figure 3B). The top ten countries by publication volume are the USA, China, Italy, Canada, Germany, France, Japan, England, Spain, and Korea. The USA and Japan were the earliest to contribute to the field, whereas China’s involvement began in 2008, with a markedly rapid increase in publication rate in recent years (Figure 3C).

Country Citation Burst Analysis assesses research activity and impact in the RBD-PD domain (Figure 3D). The earliest burst began in 1998, with Luxembourg exhibiting the longest duration (1998–2014), while the most intense citation burst is observed in China (15.57), which continues. Inter-country collaboration analysis was performed for the 30 countries with more than eight co-authored publications (Figure 3E). The USA emerged as the most collaborative, demonstrating strong research partnerships with Germany, Italy, France, and Canada.

### 3.3 Analysis of institutions

**Figure 4:**
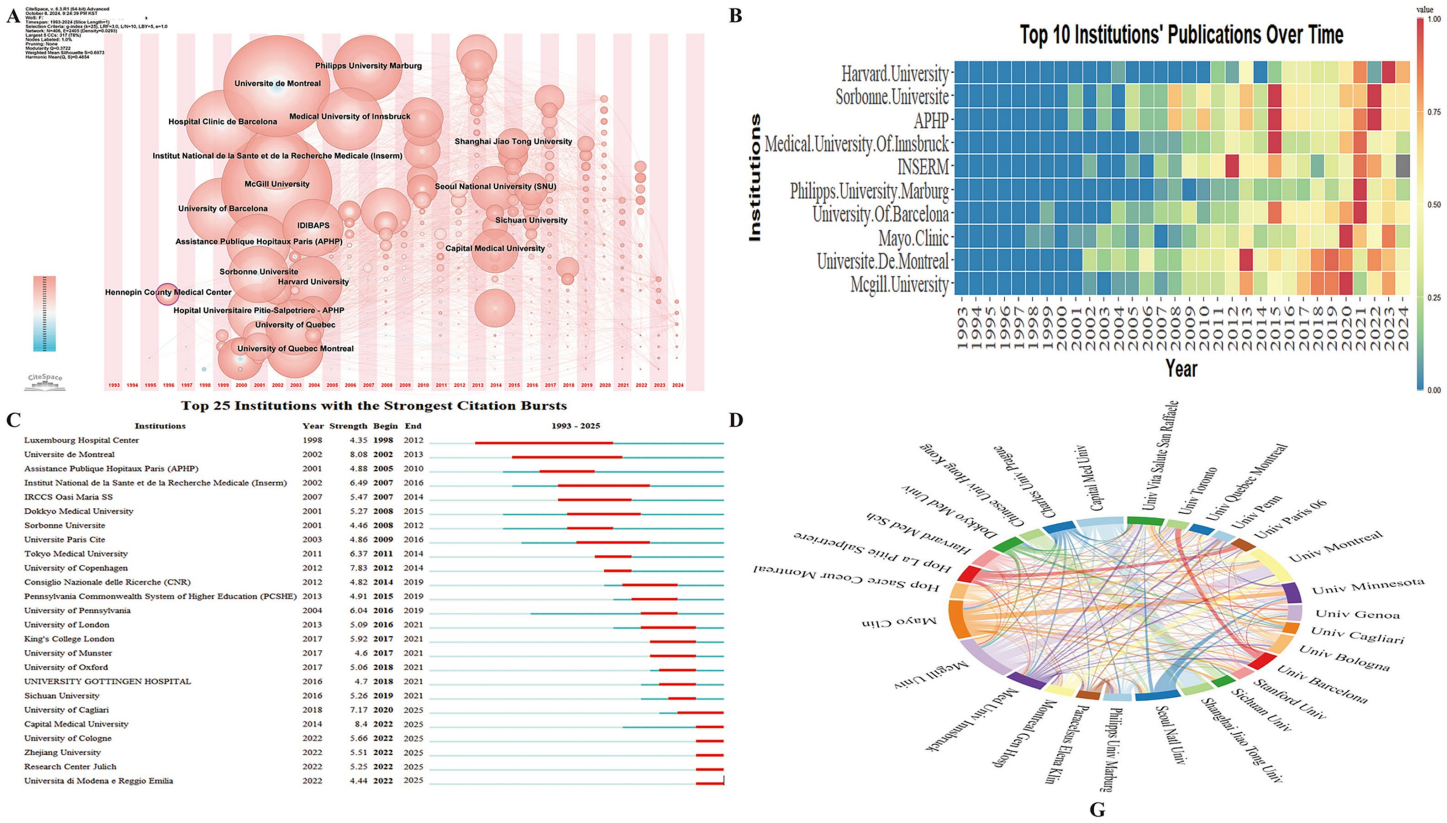
Institutional collaboration network in research on REM Sleep Behavior Disorder in Parkinson’s disease. **(A)** Co-occurrence network showing the timezone of contributing institutions. **(B)** Temporal trends in annual publication output from the top 10 most prolific institutions. **(C)** Top 25 institutions with the strongest citation bursts, highlighting periods of notable academic impact. **(D)** Collaboration network among institutions, where link thickness indicates the strength of collaboration.

Institutional analysis identifies key institutions in RBD-related PD research and examines collaboration patterns, offering insights for enhancing future cooperation and knowledge exchange. A total of 1,889 institutions have contributed to this field, with the Hennepin County Medical Center from the USA exhibiting a high centrality score (0.10), underscoring its pivotal role (Figure 4A). It is also one of the earliest institutions to engage in this research and has maintained a continuous presence.

Prominent institutions leading in publication volume include McGill University, Université de Montréal, Mayo Clinic, University of Barcelona, and Philipps University Marburg (Figure 4B). Analysis of the Top 25 Cited Institutions with the Strongest Citation Bursts (Figure 4C) shows that six institutions continue to experience significant citation activity. These active institutions are the University of Cagliari and Università di Modena e Reggio Emilia (Italy), Capital Medical University and Zhejiang University (China), and the University of Cologne and Research Center Jülich (Germany), indicating ongoing influential research efforts.

Institutional collaboration analysis was conducted for the 28 institutions with over 25 co-authored publications (Figure 4D). The strongest collaborative relationships were observed between Capital Medical University and Shanghai Jiao Tong University (China), and between McGill University and Université de Montréal (Canada). This indicates a need for expanding international collaboration to further advance research in this domain.

### 3.4 Analysis of authors

**Figure 5:**
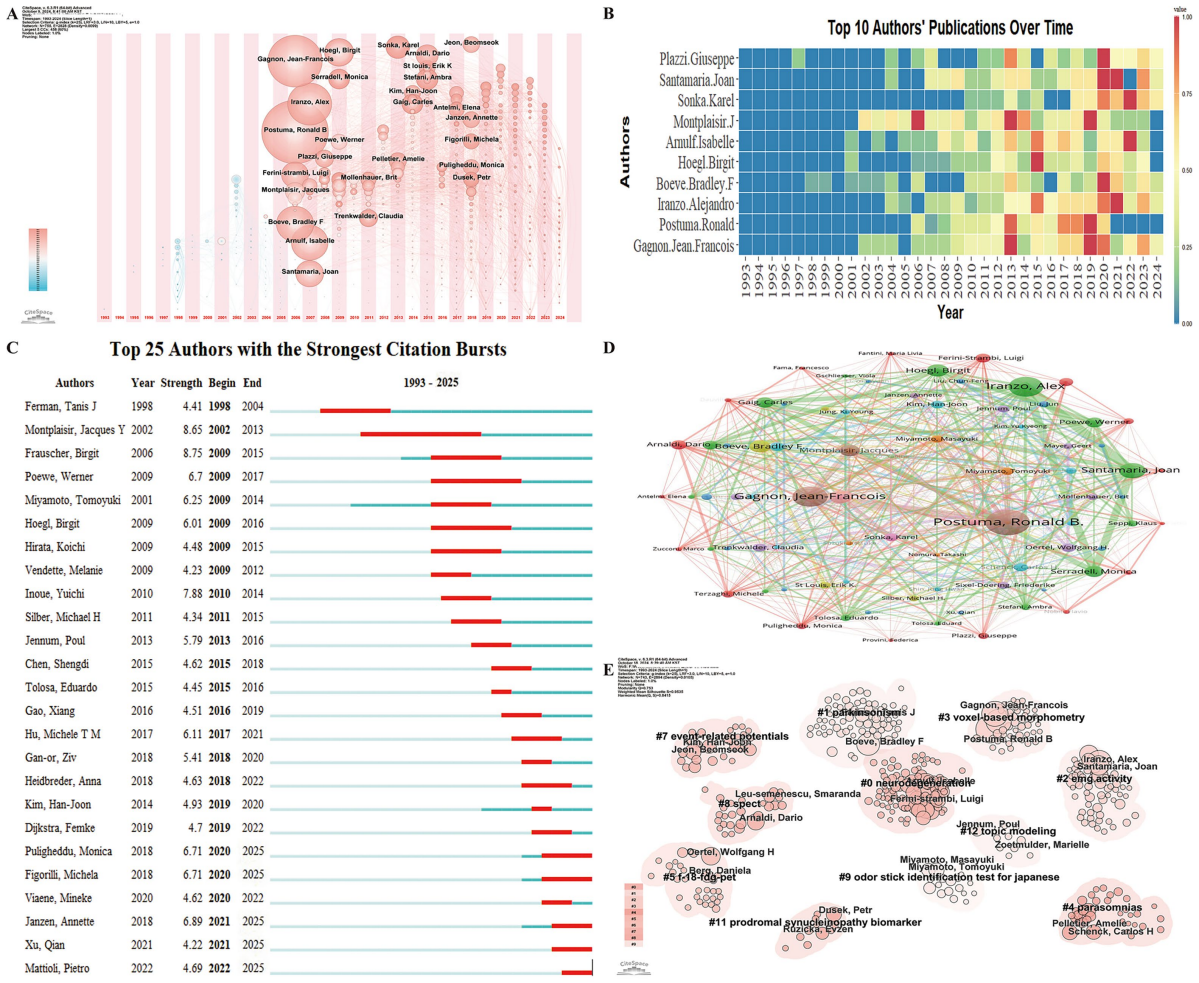
Author collaboration network in research on REM Sleep Behavior Disorder in Parkinson’s disease. **(A)** Co-occurrence network of contributing authors presented in a timezone view. **(B)** Temporal trends in annual publication output from the top 10 most prolific authors. **(C)** Top 25 authors with the strongest citation bursts, highlighting periods of intense academic influence. **(D)** Co-authorship network of contributing authors in REM Sleep Behavior Disorder research on Parkinson’s disease. Nodes are colored by cluster membership to reflect collaborative patterns, with node size corresponding to the frequency of co-authorship and links indicating co-authored publications between authors. **(E)** Author research clusters formed based on author collaboration and the similarity of their research.

Author analysis provides insights into the leading experts in RBD-related PD research, offering a reference point for potential collaborations. A total of 6,692 authors have contributed to the field, with an international collaboration rate of 26.85%. The top 10 most prolific authors include Gagnon Jean-François, Postuma Ronald, and Montplaisir Jacques Y from Canada; Iranzo Alejandro and Santamaria Joan from Spain; Boeve Bradley F from the USA; Hoegl Birgit from Austria; Arnulf Isabelle from France; Šonka Karel from the Czech Republic; and Plazzi Giuseppe from Italy. Most of these researchers began focusing on this area between 2006 and 2009 and have continued to engage in substantial, ongoing research (Figures 5A,B).

The Top 25 Cited Authors with the Strongest Citation Bursts analysis identified Frauscher Birgit as having the strongest burst strength, while Montplaisir Jacques Y exhibited the longest citation burst duration (Figure 5C). Several authors showed ongoing citation bursts, indicating continued influence. A co-authorship analysis was conducted on 110 authors with more than 10 co-occurrences (Figure 5D). These authors were grouped into 11 clusters based on collaboration intensity and research focus. Figure 5E illustrates the key authors within each research direction, highlighting their roles in advancing RBD-related PD studies.

### 3.5 Analysis of journals

**Figure 6:**
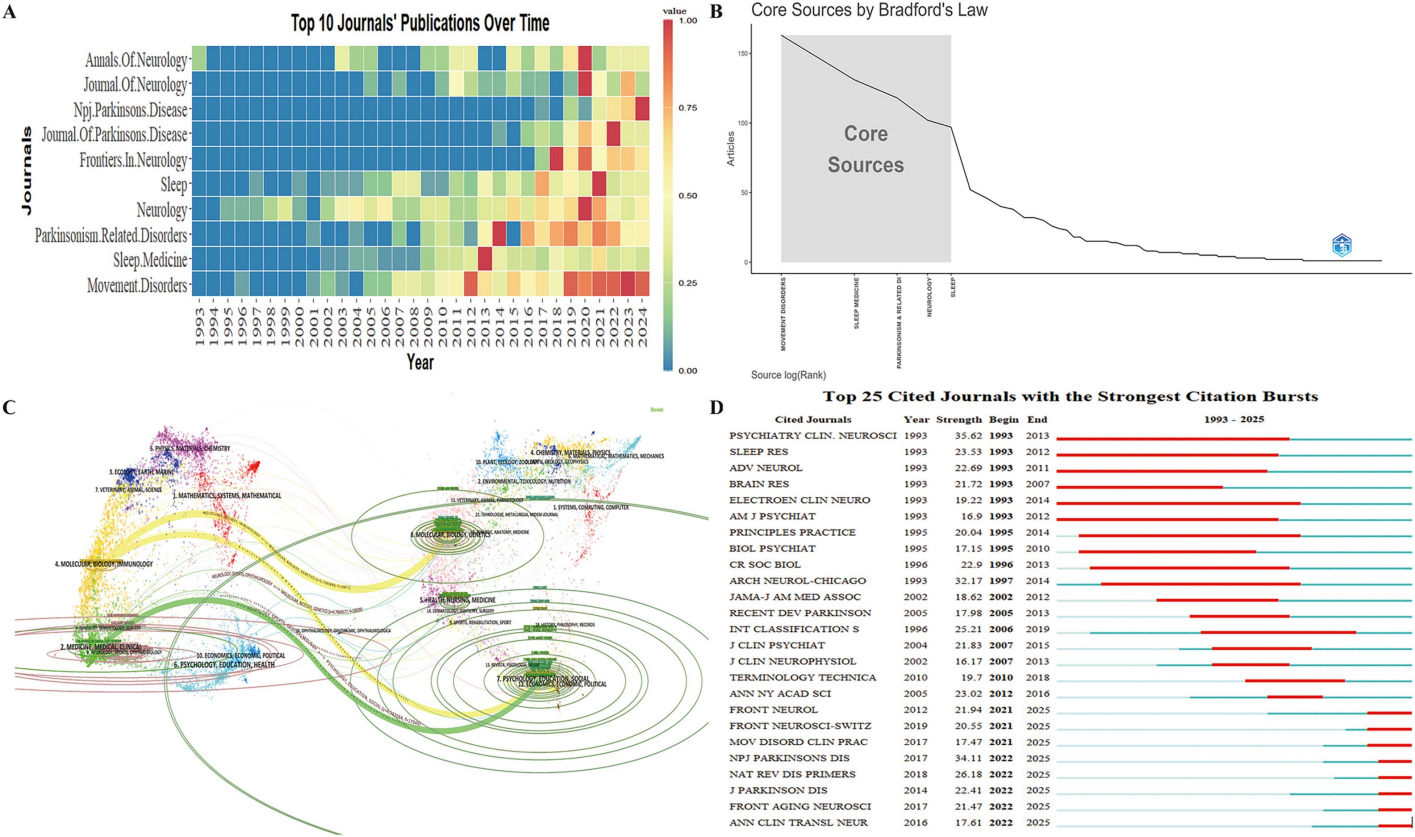
Academic journals related to REM Sleep Behavior Disorder in Parkinson’s disease. **(A)** Temporal trends in annual publication output from the top 10 most prolific academic journals. **(B)** Bradford’s Law according to the academic journals. **(C)** Dual-map overlay of the citation landscape in REM Sleep Behavior Disorder research within Parkinson’s disease. On the left, clusters represent citing journal groups, while on the right, clusters represent the most frequently cited journals. Colored lines connecting the two maps illustrate the citation relationships between citing and cited journal clusters. **(D)** Top 25 cited journals with the strongest citation bursts.

Journal analysis identifies key journals in RBD-related PD research, aiding researchers in tracking trends, staying informed, and selecting suitable publication venues. A total of 297 journals have published work in this area. The yearly publication output of the top 10 journals is shown in Figure 6A. According to Bradford’s Law Core Sources (Figure 6B), the most influential journals are *Movement Disorders*, *Sleep Medicine*, *Parkinsonism Related Disorders*, *Neurology*, and *Sleep*, which together account for 35.34% of the total publications (1,729). Notably, four of these journals are ranked in the Journal Citation Reports (JCR) Q1 category, reflecting their high research quality.

The journal overlay visualization (Figure 6C) illustrates the developmental pathways and trends in RBD-related PD research. Given the database integration constraints, the dual-map overlay of journals was generated using WoSCC data. The Citing Journals primarily from fields such as medicine, clinical sciences, molecular biology, immunology, neurology, and sports science. The Cited Journals mainly belong to psychology, education, social sciences, molecular biology, and genetics. The three thickest citation paths indicate a shift in research focus from molecular biology and psychosocial education toward molecular immunology, clinical medicine, and neurology-related sports sciences. Citation burst analysis identified journals with strong historical and ongoing influence, including Frontiers in Aging Neuroscience and NPJ Parkinson’s disease (Figure 6D).

### 3.6 Analysis of reference

Reference analysis uncovers the knowledge structure, research hotspots, interdisciplinary connections, and academic influence within a field, offering insights into its historical and current status while guiding future research directions and strategic decisions (Chen et al., 2010). In the domain of RBD-related PD research, the Top 10 Highly Cited Papers (Supplementary Table S1; Wirdefeldt et al., 2011; Stiasny-Kolster et al., 2007; Seppi et al., 2011; Seppi et al., 2019; Schenck et al., 1996; Schenck et al., 2013; McKeith et al., 2005; McKeith et al., 2017; Connolly and Lang, 2014; Armstrong and Okun, 2020) primarily focused on RBD diagnosis, management, and treatment of RBD, as well as its association with PD and dementia with Lewy bodies (DLB). These studies underscore the role of RBD as a precursor symptom for PD and DLB, highlighting the significance of early detection and intervention in mitigating the progression of PD pathology.

**Figure 7:**
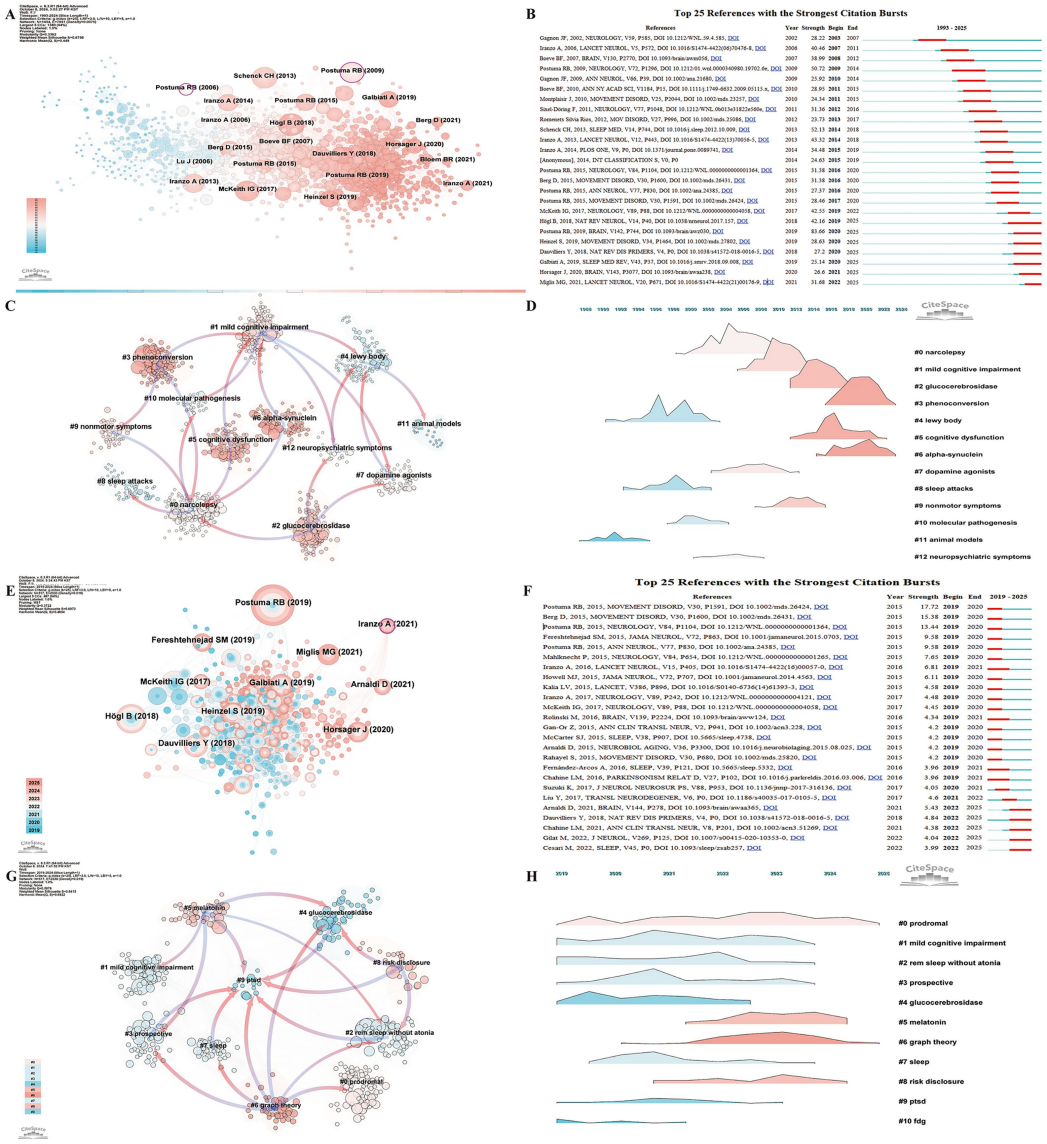
Reference analysis in REM Sleep Behavior Disorder research in Parkinson’s disease. **(A)** Co-citation network visualization from 1993 to 2025, where node size represents citation frequency. The transition from pink to blue in node color reflects the temporal distribution of publications, with pink denoting more recent works. **(B)** Top 25 references with the strongest citation bursts from 1993 to 2025. **(C)** Reference clustering based on semantic similarity from 1993 to 2025, with pink arrows illustrating the primary flow of citations within each cluster. **(D)** Landscape map of reference clusters from 1993 to 2025. **(E)** Co-citation network visualization from 2019 to 2025, where node size represents citation frequency. **(F)** Top 25 references with the strongest citation bursts from 2019 to 2025, underscoring recent influential works. **(G)** Top 25 references with the strongest citation bursts from 2019 to 2025. **(H)** Landscape map of reference clusters from 2019 to 2025.

Co-citation analysis identified two highly influential papers by Ronald B. Postuma’s team, published in Neurology in 2006 and 2009, demonstrate high betweenness centrality (Figure 7A). The 2006 paper, “Potential Early Markers of Parkinson Disease in Idiopathic REM Sleep Behavior Disorder” (0.10) (Postuma et al., 2006), was the first to propose RBD as an early marker of prodromal PD. The 2009 study, “Quantifying the Risk of Neurodegenerative Disease in Idiopathic REM Sleep Behavior Disorder” (0.13) (Postuma et al., 2009), expanded on these findings.

The Top 25 References with the Strongest Citation Bursts from 1993 to 2025 (Figure 7B) show that the earliest burst began in 2003, with the longest sustained burst occurring between 2009 and 2014. The reference with the strongest citation burst (83.66), still ongoing, is a 2019 review by Postuma’s team, published in *Brain*, titled “Risk and Predictors of Dementia and Parkinsonism in Idiopathic REM Sleep Behavior Disorder: A Multicentre Study” (Postuma et al., 2019). Currently, seven papers are experiencing citation bursts (Postuma et al., 2019; Galbiati et al., 2019; Hogl et al., 2018; Heinzel et al., 2019; Dauvilliers et al., 2018; Horsager et al., 2020; Miglis et al., 2021), they address phenoconversion risk, biomarkers, and prodromal disease trajectories. Cluster analysis of references (Figures 7C,D) indicates that cluster #3 (phenoconversion), cluster #5 (cognitive dysfunction), and cluster #6 (alpha-synuclein) are the most recent, reflecting evolving research interests and emerging trends in the field. The 2019–2025 Top 25 References with the Strongest Citation Bursts (Figures 7E,F) indicates that five papers are experiencing ongoing citation bursts (Cesari et al., 2022; Gilat et al., 2022; Dauvilliers et al., 2018; Arnaldi et al., 2021; Chahine et al., 2021), focusing primarily on RBD diagnosis, prediction of phenoconversion, and pharmacological treatment. These studies emphasize the current state of drug therapies, advocating for larger-scale clinical trials to evaluate long-term efficacy and stressing the significance of early identification and intervention in RBD management.

Given the database integration constraints, citation burst detection and reference clustering were based solely on the WOSCC data. Reference cluster analysis (Figures 7G,H) reveals that the latest clusters are #0 (prodromal), #5 (melatonin), #6 (graph theory), and #8 (risk disclosure). This analysis not only highlights current research hotspots but also traces the evolution of these topics over time, illustrating emerging trends. From the 1993–2025 Cluster Dependency Analysis (Figure 7C), two main trends emerge: (1) The first trend shows an evolution from cluster #0 (narcolepsy) to cluster #9 (non-motor symptoms), subsequently advancing to the latest clusters #3 (phenoconversion), #5 (cognitive dysfunction), and #6 (alpha-synuclein); (2) The second trend indicates that cluster #4 (Lewy body disease) developed into cluster #1 (mild cognitive impairment) and cluster #12 (neuropsychiatric symptoms), which further evolved into the latest clusters #3 (phenoconversion), #6 (alpha-synuclein), and #2 (glucocerebrosidase). In the past 5 years, cluster #2 (glucocerebrosidase) and cluster #5 (cognitive dysfunction) have advanced into cluster #5 (melatonin) (Figure 7G). Concurrently, cluster #3 (phenoconversion) has evolved into cluster #6 (graph theory), and cluster #6 (alpha-synuclein) has transformed into cluster #8 (risk disclosure), reflecting the dynamic shifts in research priorities and methodologies within the field.

### 3.7 Hotspots and trends

**Figure 8:**
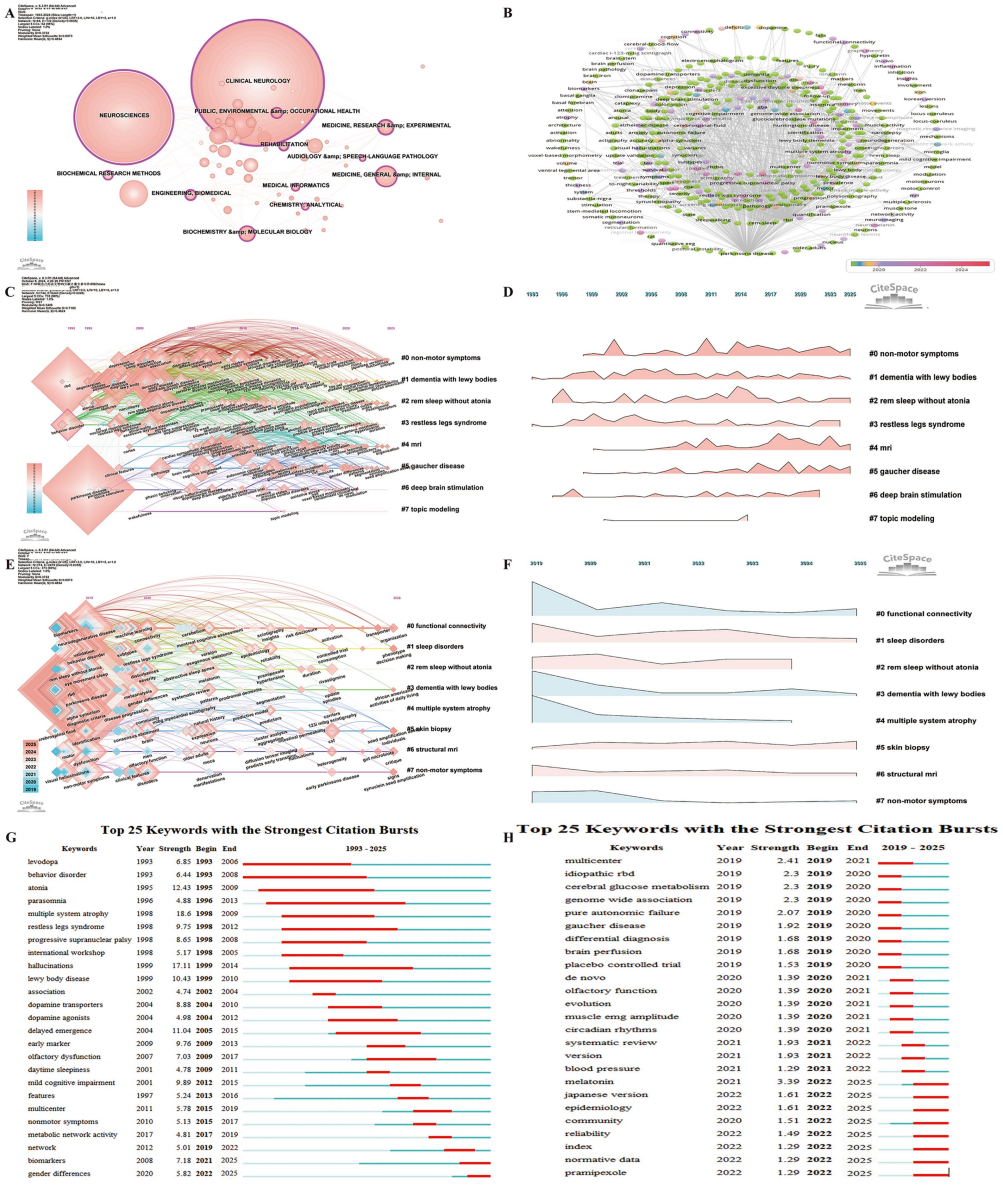
Analytical overview of research domains and key terms in the study of REM Sleep Behavior Disorder in Parkinson’s disease. **(A)** Co-occurrence network of research fields and disciplines, where node size reflects citation frequency. **(B)** Co-occurrence network of prominent keywords from 2019 to 2025, illustrating key terms driving recent research. **(C)** Timeline map of keyword clusters from 1993 to 2025, showing the evolution of research topics over time. **(D)** Landscape map of keyword clusters from 1993 to 2025. **(E)** Timeline map of keyword clusters from 2019 to 2025. **(F)** Landscape map of keyword clusters from 2019 to 2025. **(G)** Top 25 keywords with the strongest citation bursts from 1993 to 2025. **(H)** Top 25 keywords with the strongest citation bursts from 2019 to 2025.

Keyword analysis serves as a key bibliometric tool for tracking the evolution and identifying research trends in the field revealing current hotspots(Ellegaard and Wallin, 2015; Chen and Song, 2019). Category-based keyword analysis indicates that 12 categories exhibit high betweenness centrality (Figure 8A) with Neurosciences (0.82) Clinical Neurology (0.42) Engineering Biomedical (0.39) and Rehabilitation (0.24) playing key roles underscoring their central influence in RBD-related PD research. Co-occurrence analysis reveals that in addition to “Parkinson’s disease” and “RBD,” frequently occurring keywords predominantly focus on clinical manifestations pathophysiology early diagnosis and the link between RBD and cognitive impairment or neurodegenerative disease progression.

Keyword burst detection using VOSviewer was performed for keywords with more than five co-occurrences (Figure 8B). Recent keywords include fMRI MRI organization atrophytreatment outcome trial control symptoms predicts early transition mitochondrial dysfunction hypertension prodromal Parkinson disease skin biopsy clock genes and cerebrospinal fluid signaling emerging research hotspots in recent years.

CiteSpace cluster analysis of keywords from 1993 to 2025 (Figure 8C) shows that behavior disorder (0.12) and Lewy body disease (0.10) possess high betweenness centrality, indicating their key roles in PD and RBD research, aligning with the trends observed in reference analysis (Figures 7C,G). The analysis produced eight clusters, with the earliest being cluster #1 (dementia with Lewy bodies) and cluster #3 (restless legs syndrome). Cluster #1 initially centered on terms like RBD and degenerative disease, with prodromal dementia recently emerging. The latest cluster is cluster #4 (MRI), featuring new keywords such as organization, predictors, machine learning, cerebellum, and cells. The largest cluster, cluster #0 (non-motor symptoms), includes recent terms like critique and fluctuations. Active clusters include cluster #0 (non-motor symptoms), cluster #1 (dementia with Lewy bodies), cluster #2 (REM sleep without atonia), cluster #4 (MRI), and cluster #5 (Gaucher disease) (Figure 8D). Keyword clustering analysis for 2019–2025 (Figures 8E,F) identifies clusters such as cluster #0 (functional connectivity), cluster #5 (skin biopsy), and cluster #6 (structural MRI) as not only active but also rapidly expanding, indicating that these areas represent emerging and evolving research trends in the field.

Keyword bursts offer valuable insights into keyword trends helping detect shifts in research focus over time. Analysis of the top 25 keywords with the strongest citation bursts from 1993–2025 (Figure 8G) reveals that “levodopa” and “behavior disorder” were among the first to exhibit bursts while “parasomnia” (1996–2013) had the longest burst duration. The keyword “multiple system atrophy” showed the highest burst strength (18.6). Currently “biomarkers” and “gender differences” are still experiencing active bursts.

For the 2019–2025 period (Figure 8H), keywords such as “melatonin,” “Japanese version,” “epidemiology,” “community,” “reliability,” “index,” “normative data,” and “pramipexole” are undergoing active bursts. These terms reflect a focus on the diagnosis, epidemiology, treatment, and research methodologies related to RBD, suggesting that controlling RBD could potentially reduce or delay the onset of Parkinson’s disease, indicating their prominence as current research hotspots.

Tracing the evolution of research across different timeframes reveals a shift in focus. Early studies concentrated on the clinical features of RBD and its association with other neurodegenerative disorders, suggesting shared pathological mechanisms between RBD and these diseases. Later research shifted toward establishing RBD as a potential early marker for neurodegenerative conditions, particularly Parkinson’s disease and Lewy body dementia. The use of drugs like “levodopa” and dopamine agonists demonstrated that RBD is associated with dopamine transporter dysfunction. Additionally, international workshops on RBD facilitated global discussions, shedding light on phenomena such as hallucinations and the delayed onset of RBD, reinforcing its role as an early indicator of neurodegeneration. More recent studies have delved into the relationship between RBD and the non-motor symptoms of PD, as well as the role of biomarkers in tracking neurodegenerative disease progression. Non-motor symptoms such as olfactory dysfunction, daytime sleepiness, and mild cognitive impairment have gained prominence. Multicenter studies have expanded the understanding of RBD by uncovering aspects such as metabolic network activity and gender differences, further elucidating the relevance of biomarkers in predicting disease progression.

Collectively, the bibliometric analyses demonstrate a rapidly expanding and increasingly interdisciplinary research landscape in RBD-related Parkinson’s disease. Global collaboration has intensified, core research groups and institutions have emerged, and thematic evolution reveals a transition from descriptive clinical studies toward biomarker-driven prediction and early intervention research.

## 4 Discussion

### 4.1 Overall knowledge structure and evolution of RBD-related Parkinson’s disease research

RBD is not only a prevalent non-motor symptom of PD but also a recognized early marker for its onset (Schutz et al., 2022; Byeon, 2020; Benbir-Senel et al., 2024; Berg et al., 2021; Borghammer and Van Den Berge, 2019; Postuma et al., 2019; Galbiati et al., 2019; Baumann-Vogel et al., 2020; Xie et al., 2021). With the global aging population expanding, there is growing attention on RBD for its role in the early prevention and effective intervention of PD. This bibliometric analysis provides a systematic overview of the intellectual landscape and evolutionary trajectory of research at the intersection of RBD and PD. This study maps how scientific attention has shifted, consolidated, and diversified across disciplines, methodologies, and translational priorities over time. The rapid increase in the number of publications characterized by a strong linear growth trend, reflects the expanding recognition of RBD as a critical prodromal manifestation of synucleinopathies rather than merely a secondary non-motor symptom. Importantly, this growth trend mirrors patterns reported in previous bibliometric analyses of prodromal PD and non-motor symptoms (Shi and Yih, 2024; Zhao et al., 2024; Sun et al., 2024; Pan et al., 2024; Cinpolat and Akar, 2023), which similarly identified RBD as a central research axis linking sleep medicine, neurology, and neurodegeneration. The expansion of the field appears to coincide with a broader paradigm shift in PD research, from motor-centric models toward early, system-level disease conceptualizations.

The geographical and institutional distribution of publications reveals a highly centralized research structure. Currently, countries like the USA, Japan, and England lead RBD-related PD research, with the USA and Japan being the earliest pioneers in the field, forming the core of international collaboration networks. This pattern likely reflects the long-standing investment in sleep medicine research in the USA and the demographic pressures of population aging in Japan, which have driven large-scale longitudinal cohort studies of RBD. In contrast, China has emerged as the fastest-growing and most promising contributor in recent years, likely driven by demographic aging, increased investment in neuroscience research, and expanding participation in international collaborations. However, network analyses indicate that cross-regional collaboration remains relatively fragmented, highlighting an underdeveloped global research integration despite the field’s translational ambitions.

Co-authorship and co-citation analyses further demonstrate influential researchers and institutions. Leading institutions in this field include McGill University, Mayo Clinic, University of Barcelona, and Philipps University Marburg, while prominent researchers such as Jean-Francois Gagnon, Ronald Postuma, Bradley F. Boeve, and Birgit Hoegl have made significant contributions. The prominence of these institutions and researchers reflect the centrality of long-term prospective cohort designs and phenoconversion studies in advancing RBD research.

Co-citation and reference clustering analyses further demonstrate that the knowledge base of RBD-related PD research is organized around a number of highly influential conceptual milestones. Seminal studies by Ronald B. Postuma’s team, published in *Neurology* in 2006 and 2009 (Postuma et al., 2006; Postuma et al., 2009), were among the first to suggest that RBD may serve as an early marker of prodromal PD. Current research primarily revolves around two key themes: narcolepsy and Lewy body disease. Recent studies experiencing citation bursts emphasize themes such as *α*-synuclein pathology, cognitive dysfunction, and ethical considerations of risk disclosure, underscoring the maturation of the field from descriptive observation toward anticipatory and prognostic frameworks (Postuma et al., 2019; Galbiati et al., 2019; Hogl et al., 2018; Heinzel et al., 2019; Dauvilliers et al., 2018; Horsager et al., 2020; Miglis et al., 2021). Cluster analysis of references has identified several emerging research clusters, further underscores the methodological demands of prodromal PD research relies heavily on sustained follow-up, standardized assessments, and interdisciplinary expertise. From a bibliometric standpoint, these works function as intellectual anchors, shaping subsequent research on prodromal diagnosis, phenoconversion prediction, and translational trial design.

### 4.2 Emerging hotspots and methodological frontiers: a bibliometric interpretation

Bibliometrics aims to uncover research hotspots and development trends, providing valuable insights for scholars (Ellegaard and Wallin, 2015; Chen and Song, 2019). Keyword co-occurrence, burst detection, and timeline analyses collectively reveal a clear transition in research focus over time. Current research is primarily focused on Neurosciences, Clinical Neurology, Biomedical Engineering, Rehabilitation, and Experimental Medicine. Early research concentrated on the clinical features of RBD and its associations with neurodegenerative diseases, suggesting an exploratory phase focused on shared pathological mechanisms. Later studies shifted toward investigating RBD as an early marker for neurodegenerative disorders like PD and Lewy body disease. Recent research emphasizes the link between RBD and PD’s non-motor symptoms, effective interventions, and the role of biomarkers in neurodegenerative progression, highlighting the significance of early detection and intervention in slowing PD’s pathology. This thematic shift aligns RBD research with broader trends in neurodegenerative disease research, in which early-stage identification and risk modeling have become central objectives. The field has increasingly converged on a translational core that emphasizes objective markers capable of linking sleep-related manifestations to underlying synuclein pathology. The bibliometric evidence thus suggests not only topic diversification but also methodological maturation, with growing reliance on longitudinal designs, multimodal assessment strategies, and hypothesis-driven investigations.

#### 4.2.1 Neuroimaging as a methodological frontier in prodromal PD research

The emergence of MRI-related keywords and clusters represents one of the most prominent recent methodological frontiers identified in this analysis. Burst detection and timeline mapping indicate that neuroimaging has gained sustained attention, positioning it as a central tool for investigating neural substrates associated with RBD phenoconversion. Notably, the bibliometric prominence of MRI does not reflect consensus on specific imaging findings, but rather signals a broader shift toward network-level and systems-based approaches to neurodegeneration (Matzaras et al., 2022; Campabadal et al., 2021; Chen et al., 2022). Neuroimaging enables the investigation of structural and functional alterations that precede overt motor symptoms, thereby complementing clinical and neurophysiological assessments in prodromal PD research (Ferini-Strambi et al., 2019; García-Gomar et al., 2022; Si et al., 2022; Bae et al., 2023; Gaurav et al., 2022). The clustering of MRI-related studies alongside phenoconversion and prodromal PD themes suggests that neuroimaging is increasingly integrated into predictive research frameworks, rather than serving as a standalone diagnostic modality. This pattern parallels trends reported in bibliometric studies of PD biomarkers more broadly, where imaging techniques often function as integrative components within multi-modal risk models.

The dominance of cross-sectional designs within this cluster, as inferred from citation patterns and reference structures, further highlights a translational bottleneck. While neuroimaging is widely adopted, its longitudinal and prognostic applications remain underdeveloped (Campabadal et al., 2021). This imbalance suggests that future research will prioritize large-scale, multicenter longitudinal studies to monitor the long-term prognosis of patients with iRBD, integrating MRI findings with other biomarkers to develop multi-modal predictive models (Ye et al., 2020). In summary, the bibliometric trajectory of MRI research underscores both its growing importance and its current methodological constraints within prodromal PD research.

#### 4.2.2 Peripheral biomarkers and the rise of skin biopsy research

Another notable emerging hotspot identified through keyword bursts and reference clustering is skin biopsy–based research. The rapid ascent of this topic reflects an expanding interest in peripheral biomarkers capable of detecting *α*-synuclein pathology during the prodromal phase of PD. Bibliometrically, the positioning of skin biopsy clusters within recent timelines suggests that this approach is viewed as a promising bridge between molecular pathology and clinical risk stratification.

Rather than evaluating the diagnostic performance of specific techniques, this analysis highlights skin biopsy as a symbolic marker of the field’s broader translational ambitions (Miglis et al., 2021; Kuzkina et al., 2023; Calabresi et al., 2023; Okuda et al., 2022; Stefani et al., 2021). The appeal of minimally invasive, reproducible peripheral measures aligns with the practical requirements of large-scale screening and longitudinal monitoring in iRBD populations (Meloni et al., 2023; Al-Qassabi et al., 2021; Doppler et al., 2021). The co-occurrence of skin biopsy with themes such as “prodromal” and “risk disclosure” further suggests that ethical and clinical considerations are becoming increasingly intertwined with biomarker development. Similar patterns have been reported in bibliometric analyses of cerebrospinal fluid and molecular biomarkers in PD, where methodological innovation often precedes standardized clinical implementation (Gibbons et al., 2022; Iranzo et al., 2023; Yang et al., 2021; Kuzkina et al., 2021). Importantly, the variability observed across studies, reflected indirectly through dispersed citation patterns, indicates ongoing methodological heterogeneity. From a bibliometric perspective, this heterogeneity likely contributes to the sustained research momentum of this cluster, as optimization and validation remain active areas of investigation rather than resolved questions.

#### 4.2.3 Pharmacological research: emphasis on symptom management over disease modification

Pharmacological themes constitute a distinct thematic cluster within the RBD-related PD literature. Keyword and reference clustering analyses indicate that research attention has predominantly focused on agents such as melatonin and clonazepam, reflecting a sustained emphasis on symptom management and sleep modulation, with limited bibliometric evidence supporting disease-modifying or preventative interventions (Howell et al., 2023; Du et al., 2023; Morioka et al., 2022). Notably, the absence of strong and sustained citation bursts related to neuroprotective therapies stands in sharp contrast to the rapidly expanding literature on early detection and risk prediction. This divergence underscores a structural imbalance within the field: while diagnostic and prognostic research has advanced rapidly, therapeutic innovation has lagged behind. From a bibliometric perspective, this gap represents a critical translational challenge rather than a failure of individual interventions (Zhou et al., 2021; Vecchierini et al., 2021). While this focus addresses immediate clinical needs, it contrasts with the growing emphasis on prodromal biomarkers and phenoconversion prediction identified elsewhere in the literature (Samizadeh et al., 2023; Hu et al., 2023). The divergence between diagnostic innovation and therapeutic development highlights a critical challenge for the field: translating early detection into interventions capable of altering neurodegenerative trajectories.

#### 4.2.4 Comparison with related bibliometric studies and major reviews

To date, no bibliometric studies have specifically focused on the intersection of RBD and PD. In this context, comparison with existing bibliometric analyses of PD and prodromal neurodegeneration, as well as with major narrative and systematic reviews on RBD, provide an appropriate interpretive framework. Previous bibliometric studies on PD have consistently reported a progressive shift from clinical characterization toward biomarker-oriented, prodromal, and translational research since the mid-2010s (Shi and Yih, 2024). Our findings mirror this broader trend, as evidenced by the emergence of keywords and clusters related to phenoconversion, *α*-synuclein pathology, neuroimaging, and early risk stratification within the RBD literature. This alignment suggests that RBD research has increasingly been integrated into the conceptual framework of prodromal PD rather than developing as an isolated subfield. Major narrative and systematic reviews of RBD have emphasized its high conversion rate to synucleinopathies and its value as a natural clinical model of prodromal PD (Cinpolat and Akar, 2023). While such reviews primarily synthesize clinical and mechanistic evidence, our bibliometric analysis extends these conclusions by quantitatively demonstrating how this conceptual recognition has shaped the intellectual structure of the field. Specifically, the concentration of citations around longitudinal cohort studies and biomarker-driven research highlights a collective shift from symptom description toward predictive and translational objectives. Importantly, bibliometric mapping reveals an imbalance that is less apparent in review-based literature: diagnostic and prognostic research has expanded more rapidly than therapeutic innovation. This divergence underscores a critical translational gap between early identification of high-risk individuals and the development of interventions capable of modifying disease trajectories, a challenge that warrants increased attention in future research.

### 4.3 Limitations

While this study offers valuable insights into the research hotspots and trends in Parkinson’s disease-related RBD through bibliometric analysis, several limitations should be acknowledged. First, although both the WoSCC and Scopus were used to enhance coverage and reduce database-specific bias, publications indexed exclusively in other databases may still be underrepresented. Second, newly published articles require time to accumulate citations, and the focus on literature from the past 5 years may underrepresent emerging research areas or the contributions of early-career scholars. Ongoing efforts to monitor developments in this field are necessary. Third, only English-language publications were included, which may introduce language bias and underestimate contributions from non-English-speaking regions, especially in a rapidly expanding global research field. Lastly, bibliometric visualizations inevitably reflect the analytical frameworks adopted. While established software and standard configurations were applied, the findings should be interpreted as knowledge mappings rather than definitive representations of the field. Advances in bibliometric software could help mitigate this limitation in future studies.

## 5 Conclusion

In conclusion, this study utilizes bibliometric methods to analyze the literature on RBD-related PD, identifying key countries, institutions, authors, and journals, and mapping patterns of international collaboration. The analysis captures the field’s evolution, highlights current research hotspots, and identifies emerging trends. Since 2013, the volume of publications has grown exponentially, indicating the rising significance of RBD-related PD research. The USA, Japan, England, Germany, Canada, and France currently lead research efforts, with China’s rapid growth drawing increasing attention. Notable institutions include Mayo Clinic, Université de Montréal, and McGill University, while prominent authors such as Gagnon Jean-François, Postuma Ronald, and Iranzo Alejandro have made significant contributions. Influential journals in this field include *Movement Disorders*, *Sleep Medicine*, and *Parkinsonism Related Disorders*. The research is primarily driven by two major themes: narcolepsy and Lewy body disease. The trajectory has shifted from a focus on molecular genetics and psychosocial education to areas like molecular immunology, clinical medicine, and neurokinetics. Current trends emphasize the association between RBD and non-motor symptoms of PD, effective intervention strategies, and the role of potential biomarkers in neurodegenerative disease progression. Early identification and intervention in RBD are essential for mitigating PD pathology. This study provides an objective overview of RBD-related PD research, offering valuable insights for scholars and guiding future investigations in this evolving field.

## Data Availability

The raw data supporting the conclusions of this article will be made available by the authors, without undue reservation.
