# Supplementary material for: A cross-database bibliometric analysis of rapid eye movement sleep behavior disorder in Parkinson’s disease research
Source: Front Aging Neurosci. 2026 Jan 15;17:1744493. doi: 10.3389/fnagi.2025.1744493 (PMC12852318; doi:10.3389/fnagi.2025.1744493)
Supplement: Supplementary file 1 [file Table_1.DOCX]

**Supplementary Table S1. The Top10 High Cited Articles on REM Sleep Behavior Disorder in Parkinson's disease**

| **Rank** | **Title** | **First Author** | **Journal** | **Year** | **Nc** | **Document Type** | **DOI** |
| --- | --- | --- | --- | --- | --- | --- | --- |
| 1 | Diagnosis and management of dementia with Lewy bodies - Third report of the DLB consortium | McKeith, IG | Neurology | 2005 | 3856 | Review | 10.1212/01.wnl.0000187889.17253.b1 |
| 2 | Diagnosis and management of dementia with Lewy bodies Fourth consensus report of the DLB Consortium | McKeith, IG | Neurology | 2017 | 2498 | Review | 10.1212/WNL.0000000000004058 |
| 3 | Diagnosis and Treatment of Parkinson Disease A Review | Armstrong, MJ | Jama-Journal Of The American Medical Association | 2020 | 1429 | Review | 10.1001/jama.2019.22360 |
| 4 | Pharmacological Treatment of Parkinson Disease A Review | Connolly, BS | Jama-Journal Of The American Medical Association | 2014 | 991 | Review | 10.1001/jama.2014.3654 |
| 5 | Epidemiology and etiology of Parkinson's disease: a review of the evidence | Wirdefeldt, Karin | European Journal Of Epidemiology | 2011 | 811 | Review | 10.1007/s10654-011-9581-6 |
| 6 | Delayed emergence of a parkinsonian disorder in 38% of 29 older men initially diagnosed with idiopathic rapid eye movement sleep behavior disorder | Schenck, CH | Neurology | 1996 | 773 | Article | 10.1212/WNL.46.2.388 |
| 7 | The REM sleep behavior disorder screening questionnaire -: A new diagnostic instrument | Stiasny-Kolster, Karin | Movement Disorders | 2007 | 690 | Article | 10.1002/mds.21740 |
| 8 | The Movement Disorder Society Evidence-Based Medicine Review Update: Treatments for the Non-Motor Symptoms of Parkinson's Disease | Seppi, Klaus | Movement Disorders | 2011 | 611 | Review | 10.1002/mds.23884 |
| 9 | Update on treatments for nonmotor symptoms of Parkinson's disease-an evidence-based medicine review | Seppi, Klaus | Movement Disorders | 2019 | 564 | Review | 10.1002/mds.27602 |
| 10 | Delayed emergence of a parkinsonian disorder or dementia in 81% of older men initially diagnosed with idiopathic rapid eye movement sleep behavior disorder: a 16-year update on a previously reported series | Schenck, Carlos H | Sleep Medicine | 2013 | 555 | Article | 10.1016/j.sleep.2012.10.009 |
